# Relationship between serum lipid level and meibomian gland dysfunction subtype in Korea using propensity score matching

**DOI:** 10.1038/s41598-021-95599-y

**Published:** 2021-08-09

**Authors:** Minji Ha, Jiyun Song, Sunkyoung Park, Kyungdo Han, Ho Sik Hwang, Hyun-Seung Kim, Reiko Arita, Kyung-Sun Na

**Affiliations:** 1 Department of Ophthalmology, Yeouido St. Mary’s Hospital, College of Medicine, The Catholic University of Korea, 10, 63-ro, Yeongdeungpo-gu, Seoul, 07345 Republic of Korea; 2 Department of Statistics and Actuarial Science, Soongsil University, Seoul, Republic of Korea; 3 Itoh Clinic, Saitama, Japan; 4 Department of Ophthalmology, Keio University, Tokyo, Japan

**Keywords:** Biochemistry, Chemical biology, Endocrinology

## Abstract

To analyze the relationship between systemic lipid profile levels and meibomian gland dysfunction (MGD) subtype in Korea. The ophthalmic data of 95 eyes and the serum lipid profiles of 95 patients were reviewed. These factors were compared with those of the general population using data from the Korean National Health and Nutrition Examination Survey (KNHANES), which evaluated 2,917 subjects. Of these, the comparison group (1:5 ratio; n = 475) was selected using propensity score matching according to age and sex. In addition, we analyzed the relationship between serum lipid profile levels and MGD subtypes in MGD patients. The mean high-density lipoprotein (HDL) value of the MGD patients was significantly higher than that of the general population (P < 0.0001). Moreover, the mean low-density lipoprotein (LDL) levels of the MGD patients was significantly lower than that of the general population (P = 0.0002). However, the mean total cholesterol (TC), and triglyceride (TG) levels of the MGD patients were not significantly different from those of the general population (TC: P = 0.4282, TG: P = 0.5613). In addition, no serum lipid levels statistically differed among the MGD subtypes (TC: P = 0.7650, HDL: P = 0.2480, LDL: P = 0.3430, TG: P = 0.7030). A statistically significant increase in HDL and decrease in LDL concentration were observed in the MGD group, although there was no difference in any serum lipid level among the MGD subtypes.

## Introduction

Chemical analyses of lipids secreted from normal meibomian glands (MGs) have revealed a mixture of non-polar lipids (sterols and wax esters), as well as small numbers of polar lipids (phospholipids and glycolipids)^1,2^. Previous studies have demonstrated that meibomian gland dysfunction (MGD) patients exhibit elevated concentrations of cholesterol esters in meibum samples compared to those of healthy controls^1,2^. Although normal meibum lipids have a melting point of 30–34 °C^3^, the melting point increases to 46 °C as the viscosity increases due to elevated cholesterol concentrations^4^. Because MGs are lipid-synthesizing organs, it is plausible that altered systemic lipid metabolism could affect their physiology and structure.

Dyslipidemia is a disorder of systemic lipid metabolism that is characterized by abnormally elevated total blood cholesterol (TC), triglyceride (TG), and low-density lipoprotein (LDL) levels, and/or a reduction in the level of high-density lipoproteins (HDLs)^5^. Dyslipidemia is one of the major modifiable risk factors in cardiovascular disease, which is a major cause of death in adults^6–8^. Previous studies have suggested that dyslipidemia may be related to the development of MGD; however, the current evidence is inconclusive. Meanwhile, MGD can be divided into three categories according to whether terminal duct is obstructed or not and quantitative and qualitative characteristics of the glandular secretion; hypersecretory, hyposecretory; and obstructive types. Hypersecretory forms of MGD produce excess meibum secretion and exhibit a large volume of secretion at the lid margin. In contrast, hyposecretory or obstructive MGD is characterized by a decreased volume of meibum secretion due to glandular dropout or blockage of MG orifices, respectively^9,10^. However, no previous studies have revealed any relationship between dyslipidemia and any of the MGD subtypes in East Asian populations. Determining whether pathological concentrations of systemic lipids contribute to the various subtypes of MGD would fill a gap in our understanding of the disease. Therefore, the current study aimed to assess relationship between systemic lipid profile levels and each MGD subtype. We further compared each systemic lipid value in MGD patients with those of the general population using the nationally representative data of the Korea National Health and Nutrition Examination Survey (KNHANES). The propensity score of age and sex-matched control was used as an alternative to randomized controlled trial (RCT)^11–13^. The use of this method allows for unbiased comparison of the lipid profile between MGD patients and normal control.

## Materials and methods

### Study population

This retrospective, observational study was approved by the Institutional Review Board (IRB) of the Yeouido St. Mary’s Hospital, The Catholic University of Korea (SC20RISI0073), and was conducted according to the ethical principles outlined in the Declaration of Helsinki. Informed consent was waived by ethics committee/IRB of the Yeouido St. Mary’s Hospital, The Catholic University of Korea due to the retrospective nature of the study and because anonymized and de-identified information were used for propensity score matching. Exceptionally, we asked patients their informed consent for publication of identifying images (Fig. 2). We reviewed the charts of patients who were diagnosed with MGD by a single examiner (N.K.S) between January 2019 and October 2020. Treatment of MGD patients often includes the supplementation of an omega-3 fatty acid, which is known to alter lipid levels; therefore, baseline lipid profiles were conducted at the time of the initial visit for all MGD patients, which included assessments of HDL, LDL, TG, and TC concentrations. All ophthalmic data were also collected during the first visit to the ophthalmology department.

Patients were diagnosed with MGD via clinical examinations based on descriptions of glandular obstruction and meibum quality. Ophthalmic examinations were conducted on both eye for all MGD patients, and data from right eye was used as representative. Digital pressure over the central third of the lower eyelid was used to evaluate gland obstructions, and meibum secretion quality was assessed via slit-lamp biomicroscopy. Each patient’s lipid profile was assessed at the time of diagnosis. The exclusion criteria were as follows: (1) rheumatic, neurological, or dermatological diseases affecting the condition of the ocular surface; (2) a history of ocular surgery within one month prior to the initiation of the study; (3) any signs of ocular infection (bacterial or viral); (4) concomitant use of topical ophthalmic medications; and (5) any known history of dyslipidemia and/or the regular use of anti-hyperlipidemia drugs.

**Figure 1:**
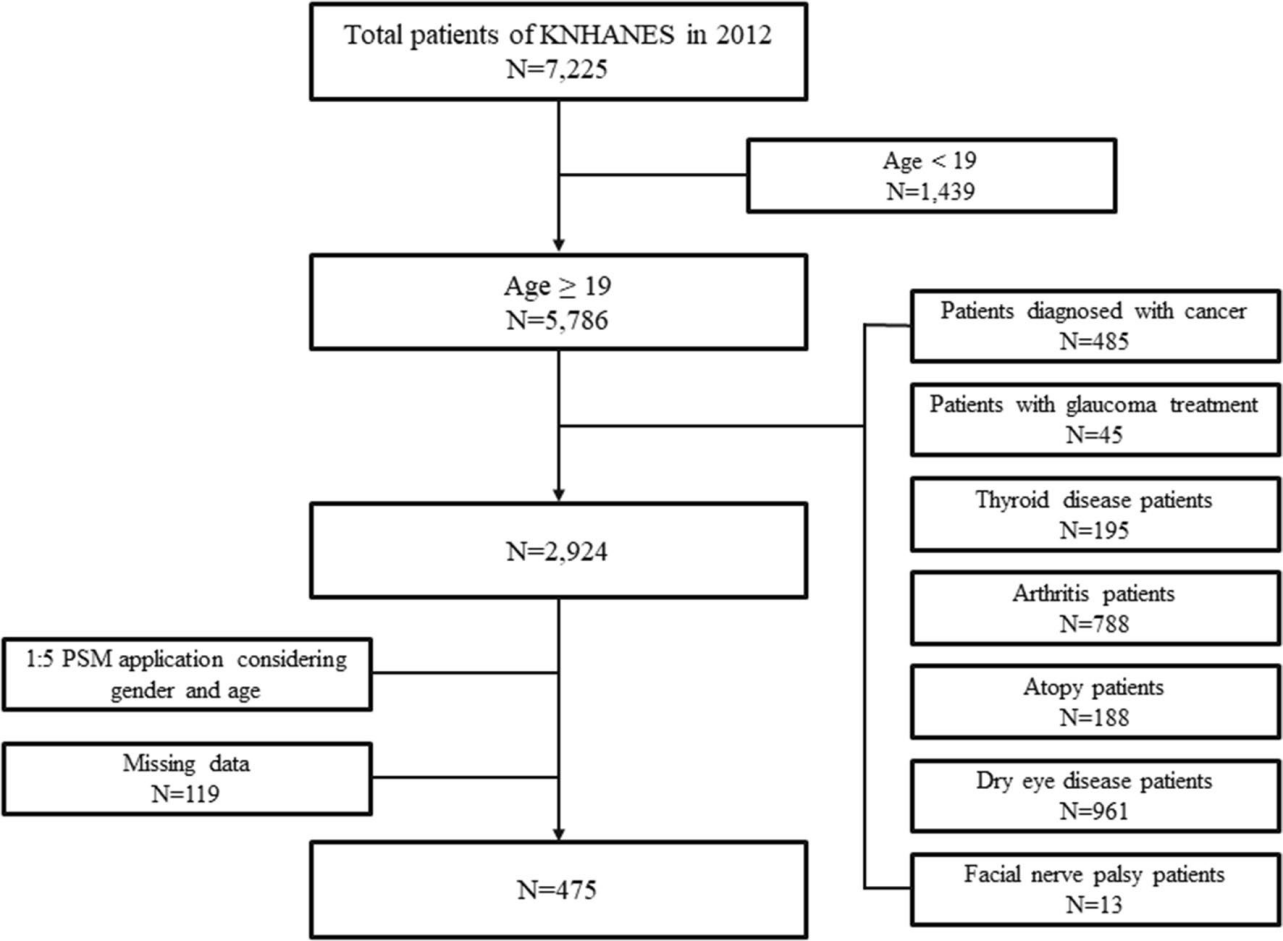
General population selection flow diagram for comparison of lipid profile between MGD and control patients.

Since there is a lack of data pertaining to ophthalmic examinations and profiling of serum lipid levels for normal controls, we used as a comparator the data of 7225 subjects in the general population that were collected in 2012 as part of the KNHANES, a survey conducted by the Division of Chronic Disease Surveillance under the guidance of the Korea Centers for Disease Control and Prevention. Among these, subjects who were aged < 19 years; received glaucoma treatment; or were diagnosed with thyroid disease, arthritis, atopy, facial nerve palsy, or dry eye disease (DED) were excluded; thus, 2917 subjects were remained. Ultimately, 475 age- and sex-matched control individuals from the general population were included in this study by applying a 1:5 propensity score matching method (Fig. 1).

The Korea National Health and Nutrition Examination Survey (KHANES), a questionnaire related to DED, was distributed, comprising the following items: “(1) Have you ever been diagnosed with DED by an ophthalmologist?” and “(2) Do your eyes tend to be dry, with a foreign body sensation including itching and burning or sandy feeling lately?” These questions could be answered as “yes” or “no.” If a participant answered “yes” to the first question, he/she was classified as “diagnosed with DED,” and if they answered “yes” to the second question, as “having symptoms of DED.” If a participant answered “no” to the questions, he/she was regarded not to have a DED associated signs or symptoms and was classified as a normal control. Subjects diagnosed with DED or as having symptoms of DED were excluded from this study. Participants who answered “do not know” were excluded from the analysis.

The age, sex, and serum lipid profiles (including TC, HDL, and TG levels) of the MGD patients were compared to those of the general population. However, LDL levels were excluded from the control group items because the 2012 KNHANES did not investigate them. Therefore, the LDL concentration was calculated using the Friedewald equation (LDL = TC − [HDL + {TG/5}]) for both group of MGD and the general population to avoid statistical error; these data are marked with an * in each table.

Peripheral blood was obtained from each subject after fasting for at least eight hours; this applied to both the MGD patients who were assessed in a single department and the normal controls in the KHANES. Serum TC, HDL, and TG concentrations were enzymatically quantified by the same methodology using a Hitachi Automatic Analyzer 7600 (Hitachi/Japan) with reagents (Pureauto SCHO-N, DAIICHI/Japan; CHOLESTEST N HDL, DAIICHI/Japan; Pureauto S TG-N, DAIICHI/Japan) at the NEODIN Medical Institute.

### Clinical examinations

Each participant underwent a thorough ophthalmic examination. The following objective tests for MGD were performed: Ocular Surface Disease Index (OSDI) questionnaire, slit-lamp examination of the ocular surface to assess the tear break-up time (TBUT) and corneal fluorescein staining, lid margin examination, MG expressibility/duct assessment, and noncontact meibography. A 5-min interval or longer was allotted between each test, except between the administration of the OSDI questionnaire and the slit-lamp examination. The OSDI questionnaire assessed symptoms on a scale ranging from 0 (no symptoms) to 100. Corneal staining was conducted using fluorescein sodium-impregnated paper strips (Haag-Sterit, Bern, Switzerland). The strips were wetted with normal saline, and diluted dye was instilled into the ocular surface. After gentle blinking, the degree of corneal staining was graded according to the Oxford scoring scheme (range of 0–5 points). TBUT, the interval between blinking and the first appearance of a dry spot on the tear film, was measured using a stopwatch.

Eight MGs in the central third area of the lower eyelid were tested for meibum expressibility and secretion following application of firm digital pressure. The meibum quality was scored according to the following criteria, which were modified from the meibum quality grading system described by Mathers et al.^14^: 0 = normal; 1 = yellow in color, without increased viscosity; 2 = yellow in color, with increased viscosity; 3 = toothpaste-like consistency; 4 = no meibum.

Meibography images were acquired using non-contact infrared meibography. The degree of MG loss was classified according to the meiboscore described by Arita et al.^15^ on a scale of 0–3, as follows: 0: no loss of MGs; 1: area loss < one-third of the total MG area; 2: area loss between one-third and two-thirds of the total MG area; 3: area loss > two-thirds of the total MG area. MGD was classified into stages ranging from 1 to 4 according to the MGD stage classification criteria published in 2011 by an international workshop on MGD^16^. Blood laboratory tests of baseline lipid profiles, including quantification of TC, TG, HDL, and LDL levels, were performed.

### Patient classification

**Figure 2:**
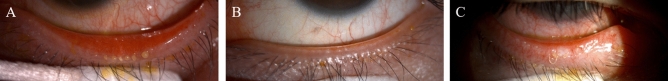
Representative photographs for each type of MGD. (**A**) Hypersecretory type of MGD. (**B**) Hyposecretory type of MGD. (**C**) Obstructive type of MGD.

MGD patients were classified into three groups according to the modified classification of Xiao et al.^17^ and Eom et al.^18^. Subjects with a meibum expressibility score greater than 5 were classified as having high-delivery MGD, whereas those with a meibum expressibility score lower than 5 were classified as having low-delivery MGD. The hypersecretory subtype belongs to the high-delivery category and a large volume of lipid with quality change was released at the inflammatory eyelid margin during compression^19^. On the other hands, the obstructive and hyposecretory subtypes of low-delivery MGD were classified according to the meibum quality and signs of lid margin inflammation. The difference between the obstructive and hyposecretory subtypes is that the obvious obstructive subtype includes subjects with inflammation and other signs of MGD pathology, while the nonobvious obstructive subtype does not^9,10,20,21^. Representative images are described in Fig. 2.

### Statistical analysis

Propensity score matching at a ratio of 1:5 was used to select the individuals comprising the normal control group from the KNHANES data to compare with the data from the MGD patients. The propensity score is the probability of receiving active treatment (Z = 1 vs. Z = 0), conditional on the observation of baseline covariates^22^. It is a balancing score; in other words, conditional on the propensity score, the distribution of measured baseline covariates is expected to be the same in treated and untreated subjects^20^. In observational studies, in order to overcome the shortcomings of the classical matching method and to minimize selection bias, a matching method using a propensity score is used. Similar to randomized, controlled trials (RCTs), propensity score methods allow one to estimate marginal, rather than conditional, measures of treatment effects^23^. The reason for this can be clearly seen for matching, stratification, and weighting, as one is comparing average outcomes between samples of treated and untreated subjects exhibiting the same distribution of observed baseline covariates^20^. After propensity score matching, t-tests were used to compare the variables (age, age distribution, sex, and lipid profile values) between the two groups.

One sample t-tests were performed to compare lipid profile values. One sample z-tests were used to compare the distribution of patients with abnormal lipid levels with those of the general population according to age range.

Clinical parameters and lipid profile values were compared among the MGD subtypes using the Kruskal–Wallis *H* test with Dunn-Bonferroni post hoc tests. Values are expressed as means and standard deviations. A p-value < 0.05 was considered statistically significant. Statistical analyses were performed using Statistical Package for the Social Sciences (SPSS) ver. 22.0 software (IBM Corp., Armonk, NY, USA) and SAS 9.1.3 (SAS Institute, Cary, NC).

## Results

Given the ethnic composition of Korean society, all of the subjects were East Asian. A total of 89 MGD patients were included in this study, with a mean age of 57.55 years ± 13.19 (range: 19–86 years), 84.21% of whom were women and 15.79% of whom were men. We stratified our patients into age brackets; 33.68% of the MGD patients were aged > 65 years, 49.47% were aged 45–64 years, and 16.84% were younger than 45 years. The mean age of the control patients in the general population selected from the KNHANES through propensity score matching (1:5) was 56.41 years ± 12.67 (range: 19–80 years); 28.21% were over 65 years old, 53.89% were 45–64 years of age, and 17.89% were under 45 years of age. Of these, 85.26% were women and 14.74% were men. There were no statistically significant differences between the two groups based on age or sex (P = 0.4282, P = 0.5613, and P = 0.7926 for mean age, age distribution, and sex, respectively) (Table 1). Additional demographic information of the general population group prior to propensity score matching is provided as supplementary information (Supplementary Table 1).

**Table 1:** Clinical characteristics of patients who applied propensity score methods (1:5).

Characteristics	MGD patients values	Control patients	P-value
No. of patients	95	475	
**Age(year)**	57.55 ± 13.19	56.41 ± 12.67	0.4282
**Age(year) range**	19–86	19–80	
**Age distribution**			0.5613
< 45(%)	16 (16.84)	85 (17.89)	
45–64(%)	47 (49.47)	256 (53.89)	
> 65(%)	32 (33.68)	134 (28.21)	
**Gender**			0.7926
Men (%)	15 (15.79)	70 (14.74)	
Women (%)	80 (84.21)	405 (85.26)	
Total cholesterol (mg/dL) (normal value: < 200)	192.96 ± 33.59	197.97 ± 38.14	0.2335
Triglyceride (mg/dL) (normal value: 34–143)	127.91 ± 75	135.4 ± 89.93	0.4468
HDL (mg/dL) (normal value: 40–60)	62.29 ± 13.17	51.42 ± 12.34	**< .0001**
LDL (mg/dL) (normal value: < 140)	105.09 ± 30.99*	119.47 ± 34*	0.0002*
**MGD stage**	2.27 ± 0.69 (1–4)		
**OSDI score**	43.65 ± 20.86 (7–93)	–	
**IOP (mmHg)**	14.48 ± 3.05 (7–21)	–	
**Oxford score**	1.40 ± 0.77 (0–3)	–	
**TBUT (s)**	2.95 ± 2.63 (1–10)	–	
**Meiboscore**	1.21 ± 0.94 (0–3)	–	

Values are presented as mean ± Standard deviation (range) or number (%).

*HDL* high-density lipoprotein, *LDL* Low-density lipoprotein, *OSDI* Ocular surface disease index, *IOP* intraocular pressure, *TUBT* tear film break-up time.

*Since KNHAES did not conduct an LDL survey in 2012, we calculated the LDL concentration using the Friedewald equation for both group of MGD and control.

**Figure 3:**
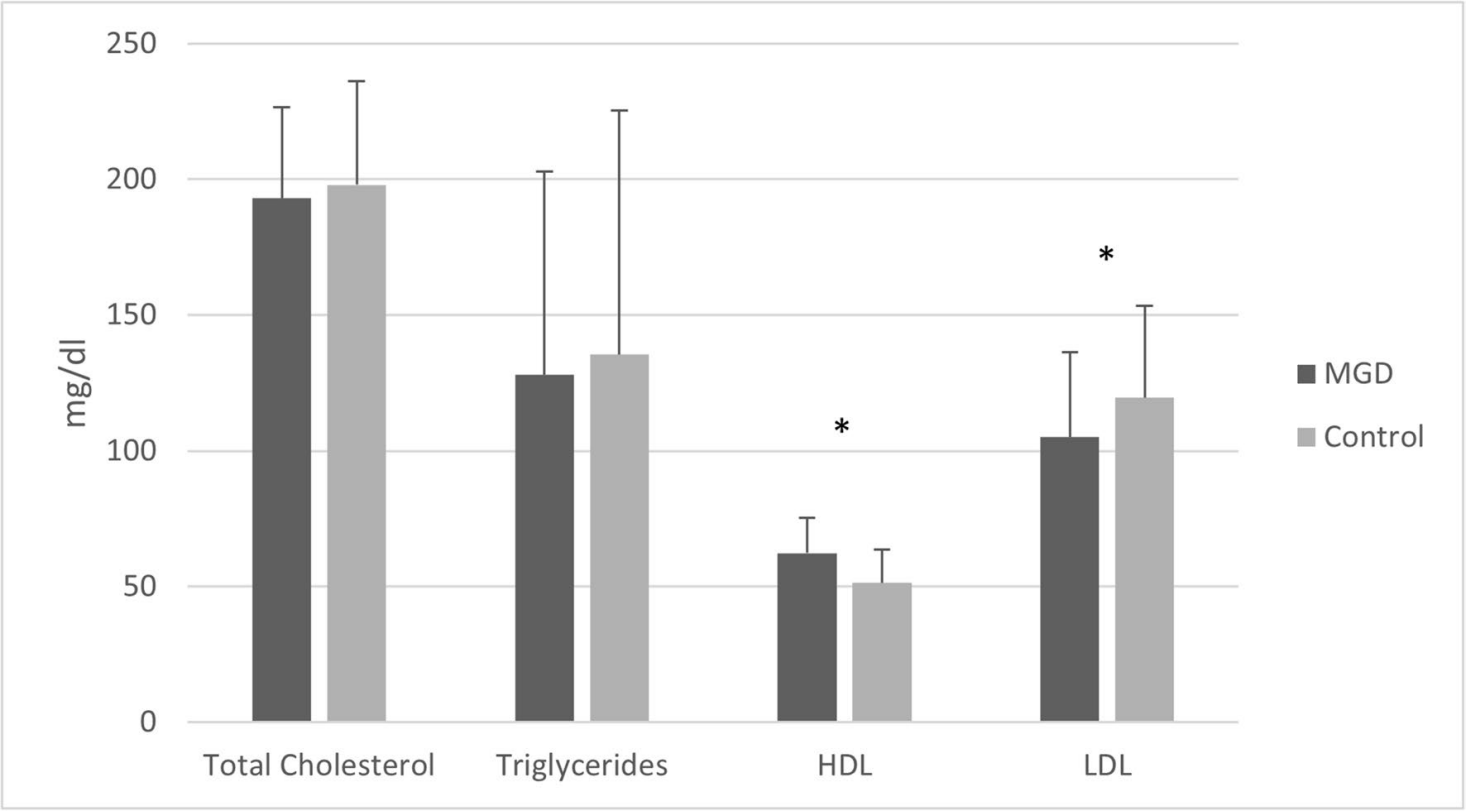
Mean value and standard deviation of lipid profile (TC, TG, HDL, LDL) of MGD and control patients. The HDL level of MGD patients was statistically significantly higher than that of the control group (P < 0.0001).

The MGD patients had high levels of HDL (62.29 ± 13.17 mg/dL). In the control patients, the HDL level was 51.42 ± 12.34 mg/dL, which was within the normal range. There was a statistically significant difference in the HDL levels between the two groups (P < 0.0001). No statistically significant differences were observed for the mean TC (P = 0.2335) and TG (P = 0.4468) levels between the two groups, and the average values of both groups were within the normal ranges. The mean LDL values of the MGD group significantly different from that of the general population (P = 0.0002), although the average values of both groups were within the normal ranges. (Table 1, Fig. 3).

In the MGD patients, the mean MGD stage was 2.27 ± 0.69 (range 1–4), the mean OSDI score was 43.65 ± 20.86 (range 7–93), the mean corneal staining grade (Oxford score) was 1.40 ± 0.77 (range 7–21), the mean TBUT was 2.95 ± 2.63 s (range 1–10 s), and the mean meiboscore of the lower eyelid was 1.21 ± 0.94 (range 0–3) (Table 1).

Based on the one sample t-tests, the mean HDL value of the MGD patients was significantly higher than that of the general population (P < 0.0001); however, the mean TC and TG level in patients with MGD was not statistically different from that of the general population (TC: P = 0.2164; TG: P = 0.331). The mean LDL values were significantly lower in the MGD group than in the general population group (LDL: P ≤ 0.0001) (Table 2).

**Table 2:** One sample t-test results of the weighted lipid profile mean value between MGD group and control group.

	Df	t-statistics	P-value
H00: total cholesterol = 196.4	94	− 1.24	0.2164
H0: triglycerides = 148.7	94	− 0.98	0.331
H0: HDL = 52.6	94	8.1	**< 0.0001**
H0: LDL = 116.7*	94*	− 4.32	< 0.0001*

*Df* degree of freedom.

*Since KNHAES did not conduct an LDL survey in 2012, we calculated the LDL concentration using the Friedewald equation for both group of MGD and control.

The bolds stand out for the “statistically significant”.

Table 3 shows the distributions of MGD patients and those in the general population with abnormal lipid profiles based on the age ranges, as compared via one sample z-tests. In the age range of 45 to 65 years, a significantly higher rate of HDL abnormalities was observed in the MGD group than in the general population, although no significant differences were observed in terms of TC or TG abnormalities between the two groups across all age ranges. On the other hand, the rate of the LDL abnormalities was significantly higher in the MGD group than in the general population in the age range of 45 to 65 years, and over 65 years old.

**Table 3:** The distribution of MGD patients with abnormal lipid profile according to the age range was compared with the average value of the general population using one sample z test.

Lipid profile	Age range	n	Event	%	p-value
Total cholesterol, > 200	< 45	16	4	25	0.5596
	45–64	47	23	48.9	0.498
	≥ 65	32	12	37.5	0.113
Triglycerides > 150	< 45	16	3	18.8	0.9718
	45–64	47	11	23.4	0.0753
	≥ 65	32	10	31.3	0.3839
HDL < 40 (40–60)	< 45	16	0	0	–
	45–64	47	3	6.4	**0.014**
	≥ 65	32	2	6.3	0.1085
LDL > 130	< 45	16*	3*	18.8*	0.6429*
	45–64	47*	9*	19.1*	0.0021*
	≥ 65	32*	7*	21.9*	0.017*

*HDL* high-density lipoprotein, *LDL* Low-density lipoprotein.

*Since KNHAES did not conduct an LDL survey in 2012, we calculated the LDL concentration using the Friedewald equation for both group of MGD and control.

Of the 95 MGD patients, the MGD subtype could be determined for 84 patients based on the meibum quality and expression test results. 11 patients were excluded as data regarding MGD was missing and subtype could not be determined. i.e., if the subjects showed high delivery state with altered meibum quality and no signs of inflammation on the eyelid margin, the subjects could not be determined in any of the subtypes. The lipid profiles of these patients were compared to determine whether they differed according to the MGD subtype. There was no statistically significant difference in lipid profile levels between any of the MGD subtypes (TC: P = 0.765, HDL: P = 0.248, LDL: P = 0.343, TG: P = 0.703). While not statistically significant, the mean HDL value was the highest in those with obstructive-type MGD. There was a statistically significant difference in the mean MGD stage among the three MGD subtypes (P = 0.000), and it was the highest in those with obstructive-type MGD (2.58 ± 0.62). The mean MG expression of patients with the hypersecretory subtype (6.89 ± 0.99) was significantly greater than that of those with the obstructive (1.31 ± 1.18) or hyposecretory (1.91 ± 1.30) subtypes (P = 0.000). The mean OSDI score was highest in the hyposecretory subtype, and there was a statistically significant difference in scores among the three subtypes (P = 0.008). The mean meiboscore was the highest in the obstructive subtype, and there was a statistically significant difference among the three subtypes (P = 0.041). The mean age, TBUT, Oxford score for corneal staining, and LLT were not statistically different among the three subtypes (Table 4).

**Table 4:** Comparison of patient characteristics and serum lipid profile according to MGD subtype.

	MGD subtype (N = 84)			P-value
	Obstructive (N = 45)	Hyposecretory (N = 11)	Hypersecretory (N = 28)	
**Age**	56.80 ± 12.34	52.36 ± 10.39	57.57 ± 15.67	0.497
**Sex**				
Male	8	2	3	0.680
Female	37	9	25	
**MGD stage**	2.58 ± 0.62	2.27 ± 0.64	1.82 ± 0.54	0.000
**OSDI score**	43.06 ± 19.04	66.81 ± 18.33	40.20 ± 20.37	0.008
**TBUT (s)**	2.76 ± 2.44	1.45 ± 0.93	2.71 ± 2.25	0.212
**Oxford stain score**	0.93 ± 0.81	0.73 ± 0.78	1.18 ± 1.02	0.304
**MG expression**	1.31 ± 1.18	1.91 ± 1.30	6.89 ± 0.99	0.000
**LLT**	81.36 ± 20.68	81.00 ± 19.14	86.407 ± 20.01	0.581
**Meiboscore**	1.49 ± 0.91	1.09 ± 0.664	0.93 ± 0.68	0.041
**Total cholesterol**	187.89 ± 34.02	192.55 ± 20.75	193.57 ± 37.27	0.765
**HDL**	65.39 ± 16.18	58.00 ± 7.68	61.11 ± 14.26	0.248
**LDL**	104.93 ± 28.48*	118.55 ± 24.63*	112.08 ± 34.28*	0.343*
**TG**	126.89 ± 58.41	108.27 ± 59.06	128.61 ± 95.64	0.703

Values are presented as mean ± Standard deviation (range) or number (%).

*MGD* meibomian gland dysfunction, *OSDI* Ocular surface disease index, *MG* meibomian gland, *LLT* lipid layer thickness, *TG* triglyceride, *HDL* high-density lipoprotein, *LDL* Low-density lipoprotein.

## Discussion

Patients with MGD exhibited increased HDL and decreased LDL levels compared with those of the controls in the normal population; this statistical significance remained despite implementation of propensity score matching methods for both age and sex. However, we observed no statistically significant differences in the lipid profile values among the three MGD subtypes. To our knowledge, this study is the first to compare serum lipid profiles according to MGD subtypes.

Also, while patients with MGD had significantly higher levels of HDL and lower levels of LDL than the age- and sex-matched controls, TC levels did not significantly differ between the two groups. The cause of this disparity in HDL and LDL levels warrants further investigation. Although HDL has cardioprotective effects, it may be a risk factor for MGD pathogenesis. Previous studies have reported a significant relationship between dyslipidemia and MGD; however, the type of cholesterol associated with MGD has varied among studies. In most studies, the TC level was higher in the MGD group than in the control group^21,24,25^. Furthermore, several studies have reported a significant association between MGD and elevated blood levels of HDL^17,18,24^. For example, Pina et al. showed in an observational, case–control, pilot study that MGD patients had a higher rate of hypercholesterolemia than the controls. In addition, MGD patients had significantly higher mean TC, LDL, and HDL levels. Moreover, based on logistic regression analysis, significantly higher levels of TC, LDL, and HDL have been found in MGD patients. Although the study conducted by Pina et al. differed from our own in terms of the age distribution, as their patient population was limited to young/middle-aged individuals (< 54 years), the high mean HDL value they reported in patients with MGD was consistent with our own study results. Dao et al. showed that patients with moderate to severe MGD (n = 66) had a higher incidence of elevated TC levels than those seen in the general population, with the component of the TC that most contributed to this increase being elevated HDL levels. However, in the comparison between the MGD group and the general population control group, their study was ambiguous about the methodology and statistical analysis related to the age- and sex-matching. In addition, since LDL generally accounts for most of the TC, it is difficult to conclude that the cause of the increase in TC observed in the MGD group resulted from the elevated HDL level.

This is the first study in which serum lipid profile levels were compared among the three MGD subtypes, and we confirmed that the mean HDL value was significantly higher in obstructive MGD patients than in those with other subtypes. Recently, HDL has received attention as a potential therapeutic target for the treatment of cardiovascular disease; however, considering our research and previous findings, HDL may negatively impact MGD pathogenesis, especially in the obstructive subtype. In general, the key role of HDL as a carrier of excess cellular cholesterol in the reverse cholesterol transportation pathway is believed to be the protection it provides against the development of atherosclerosis^26^. HDL may also slow the progression of lesions by selectively decreasing the production of endothelial cell adhesion molecules that facilitate the uptake of cells into the vessel wall^27^. HDL may, however, affect meibum production in sebaceous glands (i.e., MGs) via a mechanism that is distinct from that which mediates actions on blood vessels, although this requires further investigation through lipidomic studies^28^.

Among the MGD subtypes, obstructive MGD is predominant. Likewise, in our study, obstructive-type MGD accounted for the largest proportion of patients. Obstructive MGD presents with reduced lipid secretion, combined with the production of highly viscous meibum due to duct orifice inflammation and hyperkeratinization^29^. Hypersecretory and hyposecretory MGD, on the other hand, are not characterized by the presence of severely viscous meibum. Some authors have suggested that the clinical features of hypersecretory MGD result from a damming of secretions due to partial obstruction and that this may represent an early stage of obstructive MGD^30^. Therefore, the MGD subtypes may share a common pathophysiology. In this study, although not statistically significant, the HDL level was highest in the obstructive type. There is a possibility that elevated HDL levels may contribute to an increase in the viscosity of the meibum as hyper/hypo-secretory MGD progresses to obstructive MGD, although it is unknown how the level of HDL changes in each MGD patient over a long period of time.

This study has several limitations. Firstly, this study used data from the 2012 KNHANES, in which the ophthalmic examination of DED, MGD, or blepharitis was not performed. DED was evaluated based on the questionnaire; therefore, so any examination relating DED or grading of disease severity was not performed. Moreover, excluding DED patients does not mean that the remaining subjects did not have some type of MGD or a related ocular disease, such as blepharitis, which are prevalent and underdiagnosed. And, since LDL data were not collected in 2012, the LDL levels could not be compared based on this dataset alone. Instead, we calculated LDL levels using the Friedewald equation. Secondly, the sex ratio of the patients was very different, with the proportion of women being nearly 80%. According to Arita et al.^23^, the prevalence of symptomatic MGD (based on Japanese diagnostic criteria) was higher in men than in women (males: 42.1% versus females: 27.4%, P = 0.0051), although DED had a higher prevalence in women than in men (females: 41.7% versus males: 19.5%, P < 0.0001). In clinical practice, most patients who visit the hospital are symptomatic; therefore, it is thought that many female patients with both DED and MGD were enrolled in the study. Moreover, most of the previous studies have suggested that sex was not a significant confounding factor in the association between serum lipid levels and MGD. Thirdly, we included patients over the age of 65 years in this study, and the prevalence of MGD and dyslipidemia are known to increase with age^18^. Therefore, between the MGD and control groups, we confirmed that there were significant differences between HDL and LDL levels according to age through a one sample z-test, as shown in Table 3. Fourthly, as a study with a retrospective and observational design, it is difficult to fully explain the cause-and-effect relationships between dyslipidemia and MGD. The age and sex distribution showed a great discrepancy between the groups, mostly because 44.9% are less than 45 years old, a group that is known to have less chance to have MGD and dyslipidemia. In order to overcome the limitations of this observational study between the MGD patients and normal control, we employed age and sex-matched propensity score matching using normal control patients from the KNHANES; this approach also allowed us to overcome the possibility of selection bias, similar to RCTs. The Korean population is also relatively homogenous in terms of genetic and environmental variability, with a single race and a common climate and food culture. Thus, these potential confounding factors were unlikely to have affected the results, suggesting a very plausible association between MGD and serum lipid levels.

## Conclusion

Our results suggest that MGD patients exhibit a higher degree of HDL and lower LDL in their serum lipid profiles than individuals without MGD, although it was confirmed that there were no differences in serum lipid levels among the MGD subtypes. A large, prospective study is needed to control for additional variables to validate the possible statistically significant relationship between serum lipid levels and MGD. Further lipidomic studies should also be conducted to justify the early screening of MGD patients for the presence of dyslipidemia.

## Supplementary Information

Supplementary Table 1.
