# Supplementary material for: Relationship between serum lipid level and meibomian gland dysfunction subtype in Korea using propensity score matching
Source: Sci Rep. 2021 Aug 9;11:16102. doi: 10.1038/s41598-021-95599-y (PMC8352992; doi:10.1038/s41598-021-95599-y)
Supplement: Supplementary file 1 — Supplementary Table 1. [file 41598_2021_95599_MOESM1_ESM.docx]

**Supplement Table 1. Clinical characteristics of patients for pre-PSM.**

| **Characteristics** | **MGD patients** | **Control patients** | **P-value** |
| --- | --- | --- | --- |
|  | **values** | |  |
| **No. of patients** | 95 | 2917 | **<.0001** |
| **Age(year)** | 57.55±13.19 | 47.98±15.69 | **<.0001** |
| **Age(year) range** | 19-86 | 19-80 |  |
| **Age distribution** |  | | **<.0001** |
| <45(%) | 16(16.84) | 1311(44.94) |  |
| 45-64(%) | 47(49.47) | 1086(37.23) |  |
| >65(%) | 32(33.68) | 520(17.83) |  |
| **Gender** |  |  | **<.0001** |
| Men (%) | 15(15.79) | 1485(50.91) |  |
| Women (%) | 80(84.21) | 1432(49.09) |  |
| **Lipid profile** | | |  |
| Total cholesterol(mg/dL)  (normal value: < 200) | 192.96±33.59 | 189.5±36.49 | 0.362 |
| Triglyceride(mg/dL)  (normal value: 34-143) | 127.91±75 | 129.52±89.09 | 0.8611 |
| HDL(mg/dL)  (normal value: 40-60) | 62.29±13.17 | 50.27±12.31 | **<.0001** |
| LDL(mg/dL)  (normal value: <140) | 105.09±30.99^*^ | 113.32±32.91^*^ | **0.0164**^*^ |

Values are presented as mean $\pm$ standard deviation (range) or number (%).

HDL= high-density lipoprotein, LDL= Low-density lipoprotein

OSDI = Ocular surface disease index, IOP = intraocular pressure, TUBT= tear film break-up time

* Since KNHAES did not conduct an LDL survey in 2012, we calculated the LDL concentration using the Friedewald equation for both group of MGD and control.
